# Supplementary material for: HIVprotI: an integrated web based platform for prediction and design of HIV proteins inhibitors
Source: J Cheminform. 2018 Mar 9;10:12. doi: 10.1186/s13321-018-0266-y (PMC5845081; doi:10.1186/s13321-018-0266-y)
Supplement: Supplementary file 2 — Additional file 2. Source code of HIVProtI web server. [file 13321_2018_266_MOESM2_ESM.zip › HIVprotI_Source-code/hivprotI/weka-3-6-6/doc/weka/classifiers/bayes/net/ADNode.html]

ADNode


---


|  |  |  |
| --- | --- | --- |
| **Overview** \| **Package** \| **Class** \| **Tree** \| **Deprecated** \| **Index** \| **Help** \| **Weka's home** | |  |
| PREV CLASS   **NEXT CLASS** | **FRAMES**    **NO FRAMES**     **All Classes** |  |
| SUMMARY: NESTED \| FIELD \| CONSTR \| METHOD | DETAIL: FIELD \| CONSTR \| METHOD |  |


---


## weka.classifiers.bayes.net Class ADNode

```
java.lang.Object
  weka.classifiers.bayes.net.ADNode
```

**All Implemented Interfaces:**: java.io.Serializable, RevisionHandler, TechnicalInformationHandler

---

``` public class ADNode extends java.lang.Object implements java.io.Serializable, TechnicalInformationHandler, RevisionHandler ```

The ADNode class implements the ADTree datastructure which increases
the speed with which sub-contingency tables can be constructed from
a data set in an Instances object. For details, see:


Andrew W. Moore, Mary S. Lee (1998). Cached Sufficient Statistics for Efficient Machine Learning with Large Datasets. Journal of Artificial Intelligence Research. 8:67-91.


BibTeX:

```
 @article{Moore1998,
    author = {Andrew W. Moore and Mary S. Lee},
    journal = {Journal of Artificial Intelligence Research},
    pages = {67-91},
    title = {Cached Sufficient Statistics for Efficient Machine Learning with Large Datasets},
    volume = {8},
    year = {1998}
 }
```

**Version:**
:   $Revision: 1.7 $

**Author:**
:   Remco Bouckaert (rrb@xm.co.nz)

**See Also:**: Serialized Form

---

| **Field Summary** | |
| --- | --- |
| `Instance[]` | `m_Instances`             list of Instance children (either m\_Instances or m\_VaryNodes is instantiated) |
| `int` | `m_nCount`             count |
| `int` | `m_nStartNode`             first node in VaryNode array |
| `VaryNode[]` | `m_VaryNodes`             list of VaryNode children |


**Constructor Summary**

`ADNode()`
  
          Creates new ADNode


| **Method Summary** | |
| --- | --- |
| `void` | `getCounts(int[] nCounts, int[] nNodes, int[] nOffsets, int iNode, int iOffset, boolean bSubstract)`             get counts for specific instantiation of a set of nodes |
| `java.lang.String` | `getRevision()`             Returns the revision string. |
| `TechnicalInformation` | `getTechnicalInformation()`             Returns an instance of a TechnicalInformation object, containing detailed information about the technical background of this class, e.g., paper reference or book this class is based on. |
| `static void` | `main(java.lang.String[] argv)`             for testing only |
| `static ADNode` | `makeADTree(Instances instances)`             create AD tree from set of instances |
| `static ADNode` | `makeADTree(int iNode, FastVector nRecords, Instances instances)`             create sub tree |
| `static VaryNode` | `makeVaryNode(int iNode, FastVector nRecords, Instances instances)`             create sub tree |
| `void` | `print()`             print is used for debugging only and shows the ADTree in ASCII graphics |

**Methods inherited from class java.lang.Object**

`equals, getClass, hashCode, notify, notifyAll, toString, wait, wait, wait`

**Field Detail**

### m\_VaryNodes

```
public VaryNode[] m_VaryNodes
```

:   list of VaryNode children

---


### m\_Instances

```
public Instance[] m_Instances
```

:   list of Instance children (either m\_Instances or m\_VaryNodes is instantiated)

---


### m\_nCount

```
public int m_nCount
```

:   count

---


### m\_nStartNode

```
public int m_nStartNode
```

:   first node in VaryNode array


**Constructor Detail**

### ADNode

```
public ADNode()
```

:   Creates new ADNode


**Method Detail**

### getTechnicalInformation

```
public TechnicalInformation getTechnicalInformation()
```

:   Returns an instance of a TechnicalInformation object, containing
    detailed information about the technical background of this class,
    e.g., paper reference or book this class is based on.

    :   **Specified by:**: `getTechnicalInformation` in interface `TechnicalInformationHandler`
    :   **Returns:**: the technical information about this class

---


### makeVaryNode

```
public static VaryNode makeVaryNode(int iNode,
                                    FastVector nRecords,
                                    Instances instances)
```

:   create sub tree

    :   **Parameters:**: `iNode` - index of the lowest node in the tree: `nRecords` - set of records in instances to be considered: `instances` - data set **Returns:**: VaryNode representing part of an ADTree

---


### makeADTree

```
public static ADNode makeADTree(int iNode,
                                FastVector nRecords,
                                Instances instances)
```

:   create sub tree

    :   **Parameters:**: `iNode` - index of the lowest node in the tree: `nRecords` - set of records in instances to be considered: `instances` - data set **Returns:**: ADNode representing an ADTree

---


### makeADTree

```
public static ADNode makeADTree(Instances instances)
```

:   create AD tree from set of instances

    :   **Parameters:**: `instances` - data set **Returns:**: ADNode representing an ADTree

---


### getCounts

```
public void getCounts(int[] nCounts,
                      int[] nNodes,
                      int[] nOffsets,
                      int iNode,
                      int iOffset,
                      boolean bSubstract)
```

:   get counts for specific instantiation of a set of nodes

    :   **Parameters:**: `nCounts` - - array for storing counts: `nNodes` - - array of node indexes: `nOffsets` - - offset for nodes in nNodes in nCounts: `iNode` - - index into nNode indicating current node: `iOffset` - - Offset into nCounts due to nodes below iNode: `bSubstract` - - indicate whether counts should be added or substracted

---


### print

```
public void print()
```

:   print is used for debugging only and shows the ADTree in ASCII graphics

---


### main

```
public static void main(java.lang.String[] argv)
```

:   for testing only

    :   **Parameters:**: `argv` - the commandline options

---


### getRevision

```
public java.lang.String getRevision()
```

:   Returns the revision string.

    :   **Specified by:**: `getRevision` in interface `RevisionHandler`
    :   **Returns:**: the revision


---


|  |  |  |
| --- | --- | --- |
| **Overview** \| **Package** \| **Class** \| **Tree** \| **Deprecated** \| **Index** \| **Help** \| **Weka's home** | |  |
| PREV CLASS   **NEXT CLASS** | **FRAMES**    **NO FRAMES**     **All Classes** |  |
| SUMMARY: NESTED \| FIELD \| CONSTR \| METHOD | DETAIL: FIELD \| CONSTR \| METHOD |  |


---
